# Death of Dr. Joseph A. Eve

**Published:** 1886-02-20

**Authors:** 


### DEATH OF DR. JOSEPH A. EVE.

Dr. Joseph A. Eve, the old and honored Professor of Obstetrics in the Georgia Medical College, recently died at his home in Augusta. As far back as 1828 he was a practicing physician in that city, and was chosen a Professor of the Augusta school at its organization. He was an able writer and faithful teacher, and became very eminent and popular in his particular branch of Medical science. He was remarkable for his great kindness and benevolence of character, and was in all respects a good man and a true physician.

It is understood that Dr. Eve had for some time been engaged in preparing a work embodying his experience in Obstetric and Gynecological Practice, which we presume will be published. Anything from his pen on these subjects will, doubtless, be eagerly sought by the profession in the South, among whom are to be found many who, as pupils of the Georgia Medical College, know well his ability as an obstetrician, and cherish a warm regard for his memory.

